# Performance of the Plusoptix A09 Photoscreener in Detecting Amblyopia Risk Factors in Chinese Children Attending an Eye Clinic

**DOI:** 10.1371/journal.pone.0126052

**Published:** 2015-06-01

**Authors:** Xiao-Ran Yan, Wan-Zhen Jiao, Zhi-Wei Li, Wen-Wen Xu, Feng-Jiao Li, Li-Hua Wang

**Affiliations:** Department of Ophthalmology, Shandong Provincial Hospital affiliated to Shandong University, Jinan, Shandong, China; The Ohio State University, Center for Cognitive and Brain Sciences, Center for Cognitive and Behavioral Brain Imaging, UNITED STATES

## Abstract

**Purpose:**

To assess the accuracy of the Plusoptix A09 photoscreener in detecting amblyopia risk factors in children and determine referral criteria when using Plusoptix A09 for a large-scale vision screening.

**Methods:**

Pediatric patients attending our eye clinic underwent a comprehensive ophthalmic examination that included photorefraction, orthoptic examination, anterior segment assessment, fundus examination and cycloplegic retinoscopy. The measurements were collected for statistical analyses.

**Results:**

One hundred and seventy-eight children (mean age ± SD: 6.2±2.4 years, range: 2.2 to 14.1 years) were included in the study. The mean spherical equivalent (SE) obtained using Plusoptix A09 (P_SE_) was 0.57 D lower than that obtained from cycloplegic retinoscopy (CR_SE_) (*P * = 0.00). However, there was no statistically significant difference of Jackson cross cylinder J_0_ and J_45_ between Plusoptix A09 (P_J_) and cycloplegic retinoscopy (CR_J_) (*P* = 0.14, *P* = 0.26). The relationship of SE obtained from Plusoptix A09 and SE obtained from cycloplegic retinoscopy was presented as the equation: CR_SE_ = 0.358 + 0.776 P_SE_ + 0.064 P_SE_^2^ + 0.011 P_SE_^3^. Based on the Receiver Operating Characteristic (ROC) curve, the Plusoptix A09 had an overall sensitivity of 94.9% and specificity of 67.5% for detecting refractive amblyopia risk factors. The sensitivity and specificity of the Plusoptix A09 for detection of strabismus were 40.7% and 98.3%, respectively; detection of amblyopia and/or strabismus was 84.7% and 63.2%, respectively.

**Conclusions:**

The Plusoptix A09 photoscreener underestimated hyperopia and overestimated myopia according to SE when compared with cycloplegic retinoscopy. The accuracy of the Plusoptix A09 in detecting amblyopia risk factors in children could be improved by the regression equation and optimized criteria for refractive amblyopia risk factors developed in the present study. Moreover, the Plusoptix A09 photoscreener is not suitable for a large-scale strabismus screening when it is applied solely.

## Introduction

Amblyopia is the most common visual disorder in children with an estimated worldwide prevalence of 2–5% [1]. Amblyopia not only results in visual impairment or blindness, but also has significant effects on children’s school performances and mental health. Significant refractive errors, strabismus, anisometropia, congenital cataract and ptosis are important amblyopia risk factors [2]. Previous studies have indicated that visual impairment or blindness caused by amblyopia are avoidable by means of early intervention [3–5]. Moreover, the optimal time for amblyopia treatment was demonstrated for children younger than seven years old [6]. Therefore, early detection is of great importance for treating amlyopia. Early pediatric vision screening is recommended by the American Academy of Pediatrics, the American Association of Pediatric Ophthalmology and Strabismus (AAPOS) and the US Preventive Services Task Force [7, 8]. However, the procedures and details of vision screening are inconsistent worldwide. Some studies tended to use photorefraction for early detection of amblyopia risk factors in children’s vision screening. This screening approach was based on the evidence that noncycloplegic photorefraction had acceptable accuracy and advantages of speed and portability when compared with cycloplegic retinoscopy [9–11]. On the contrary, others regarded photorefraction without cycloplegia as unreliable because of poor accuracy and limited range of refractive errors [12, 13]. A series of the Plusoptix photoscreeners (Plusoptix GmbH, Nuremberg, Germany) are newly designed photorefraction tools for vision screening in children and are approved by the US Food and Drug Administration (FDA) as a refractor. However, this tool has not been widely employed in the pediatric primary health care program of Chinese cities. In addition, although there are some reports of the sensitivity and specificity of the Plusoptix S04 or S08 for detecting amblyopia risk factors [14–23], data from the newest version, the Plusoptix A09, are still scarce.

The purpose of the present study was to evaluate the accuracy of the Pluoptix A09 photoscreener in detecting amblyopia risk factors in children. Additionally, we validated the Plusoptix A09 photoscreener in a Chinese population to determine the Plusoptix referral criteria when the instrument is employed for a large-scale vision screening.

## Materials and Methods

### Patients

The present study was approved by the Medical Ethical Committee of Shandong Provincial Hospital affiliated to Shandong University and was in accordance with the 1975 Declaration of Helsinki. Written, informed consent was provided by the parents of the pediatric patients.

Pediatric patients attending the clinic of the Ophthalmology Department of Provincial Hospital affiliated to Shandong University during September 2012 to March 2013 were included in the study. A comprehensive ophthalmic examination was carried out in the following order: photorefraction using the Plusoptix A09 photoscreener without cycloplegia, orthoptic examination with prism alternative and cover test, anterior segment assessment using slit lamp, fundus examination and cycloplegic retinoscopy. Cases with retinal abnormalities, eccentric fixation and significant media opacities were excluded. All patients included in the study had steady visual fixation and cooperated well in all of the examinations.

### Methods

The Plusoptix A09 photoscreener was placed at a distance of one meter in front of the patient in a darkroom and operated by a trained nurse. The fixation target of the instrument was designed as a smile face on the camera. Once pressing the start button, the smile face was automatically lighted and a warble sound could be heard to draw the child’s attention to the camera. The children were asked to gaze at the nose of the smile face on the camera during the test. Then the camera was moved slightly (within 50 mm) until green circles were evident around both pupils on the monitor screen, which was followed by automatic measurement. The results were displayed on the monitor. The Plusoptix A09 photoscreener has a spherical and cylindrical range of -7.0 D to +5.0 D. If the spherical equivalent (SE) is out of the range, the measurement value only displays “Hyperopia” or “Myopia”. Ocular misalignment ≥10° could not be measured binocularly, and was changed to a sequential monocular measurement mode. Each patient was tested twice and the average value was the final result.

In another dark room an optometrist performed cycloplegic retinoscopy (CR) at a work distance of one meter. Measurements were made following three drops of 1.0% cyclopentolate applied to both eyes of the patients during 40 to 50 minutes. The patients were asked to gaze at a red spot-like symbol on the visual acuity chart at a distance of five meters during the retinoscopy examination. The optometrist performing cycloplegic retinoscopy was masked to the measurements of the Plusoptix A09 photoscreener.

An ophthalmologist with the specialty of pediatric strabismus was responsible for determining the type and amount of the strabismus deviation without glasses for distance and near via cover-uncover test and simultaneous prism cover-uncover test.

Amblyopia was diagnosed if the best corrected visual acuity (BCVA) in one or both eyes could not be improved to more than 20/32, in the absence of organic changes, and factors such as hyperopia, myopia, anisometropia or strabismus could be the cause of amblyopia [24].

According to the guidelines of AAPOS [2], a patient who had at least one factor as follows in the comprehensive examination was considered positive for having amblyopia risk factor(s): anisometropia (spherical or cylindrical) > 1.5 D; any manifest strabismus (deviation ≥ 5 PD); hyperopia > +3.5 D in any meridian; myopia > -3.0 D in any meridian; astigmatism > 1.5 D within 10° of 90° or 180°, or > 1.0 D in oblique axis; or ptosis with ≤ 1 mm margin reflex distance.

### Data analysis

Binocular measurements were obtained for all patients. Data from right eyes were analyzed to avoid enantiomorphism bias except for the anisometropia calculation [25].

To compare the refractive measurements between the Plusoptix A09 photoscreener and cycloplegic retinoscopy, data were calculated using the following equations: SE = sphere + (cylinder/2), Jackson cross cylinder at axis 0° (J_0_) = -cylinder/2 × cos (2×axis), Jackson cross cylinder at axis 45° (J_45_) = -cylinder/2 × sin (2×axis) [26], spherical anisometropia = |sphere(left)-sphere(right)|, and cylindrical anisometropia = |cylinder(left)-cylinder(right)|.

Descriptive data were presented as mean, standard deviation and frequency. Paired t-test and curve estimation regression analysis were performed to assess the difference and quantitative relationship between the measurements obtained from the Plusoptix A09 photoscreener and those of cycloplegic retinoscopy. Bland-Altman plot was used to document the agreement of the measurements of the Plusoptix A09 photoscreener and that of cycloplegic retinoscopy. Receiver Operating Characteristic (ROC) curve was employed to select the best cutoff points related to appropriate sensitivity and specificity of the Plusoptix A09 photoscreener. All statistical analyses were performed using SPSS (version 16.0, IBM-SPSS, Chicago, Illinois, USA). *P* < 0.05 was considered as statistically significant.

## Results

**Fig 1:**
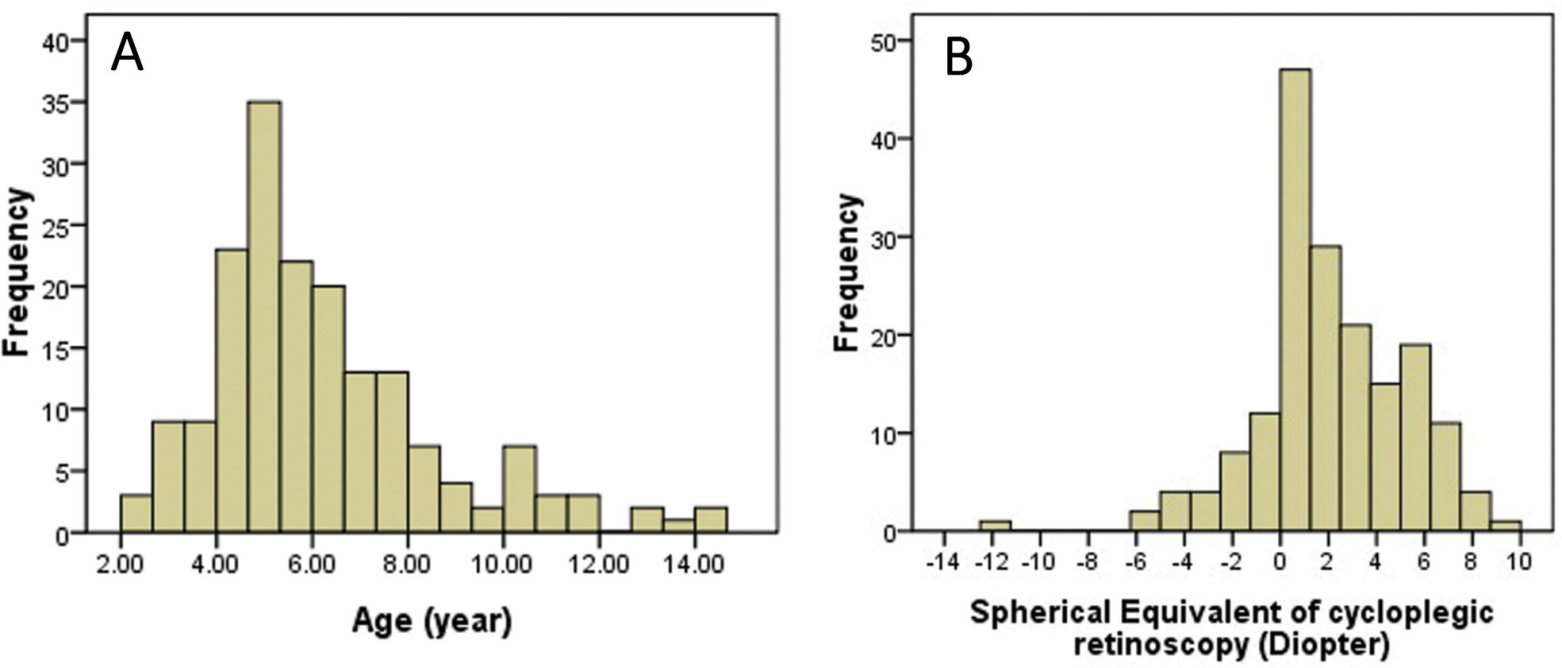
(A) Distribution of ages of the study subjects; (B) Distribution of refractive errors of the study subjects.

One hundred and seventy-eight pediatric patients (mean age ± SD: 6.2 ± 2.4 years, range, 2.2 to 14.1 years) were included in the study. There were 100 males (56.2%) and 78 females (43.8%). The visual acuities of the patients ranged from 20/200 to 20/20. Eighty-six (48.3%) patients were diagnosed with amblyopia. Sixty-three (35.4%) children were diagnosed as strabismus with a mean deviation of 27.1 ± 18.5 PD (range, 5–70 PD), of which there were heterophoria (n = 4), exotropia (n = 18), esotropia (n = 38), and vertical strabismus (n = 3). A combination of horizontal and vertical strabismus was found in two patients. The range of refractive errors (SE) measured via cycloplegic retinoscopy on all patients was from -12.0 D to +8.9 D. Hyperopia was found in 101 eyes (56.7%), of which 52 eyes had values greater than +3.5 D. Myopia was found in 27 eyes (15.2%), of which 9 eyes had values greater than -3.0 D. Emmetropia (-0.5 D to +1.0 D) was found in 50 eyes (28.1%). The distribution of ages and refractive errors is shown in Fig 1.

### Comparison between the Plusoptix A09 and cycloplegic retinoscopy

There were 28 cases with a measurement considered to be associated with “Hyperopia” or “Myopia”, due to the SEs being out of the range set for the device. These cases were excluded from the comparison of refractive measurements. Therefore, only 150 cases were included in the analysis.

The consistency of the two measurements from the Plusoptix A09 photoscreener was confirmed with the Pearson’s correlation coefficient (r = 0.95, *P* = 0.00). The mean SE and values of Jackson cross cylinder power obtained from the Plusoptix A09 photoscreener and cycloplegic retinoscopy are displayed in Table 1. There was a significant difference of SEs between the Plusoptix A09 photoscreener and cycloplegic retinoscopy (*P* = 0.00); whereas, there was no significant difference of cylinder power (J_0_ and J_45_) (*P* = 0.14 and *P* = 0.26, respectively).

**Table 1:** The mean spherical equivalent (SE) and Jackson cross cylinder power values obtained by the Plusoptix A09 and cycloplegic retinoscopy.

	Mean SE value (D)	Mean cylinder power value J_0_ (D)	Mean cylinder power value J_45_ (D)
The Plusoptix A09	1.14 ± 2.19	0.05 ± 0.63	-0.03 ± 0.70
Cycloplegic retinoscopy	1.71 ± 2.52	-0.03 ± 0.51	0.05 ± 0.58
P values *	0.00	0.14	0.26

*Paired t test

**Fig 2:**
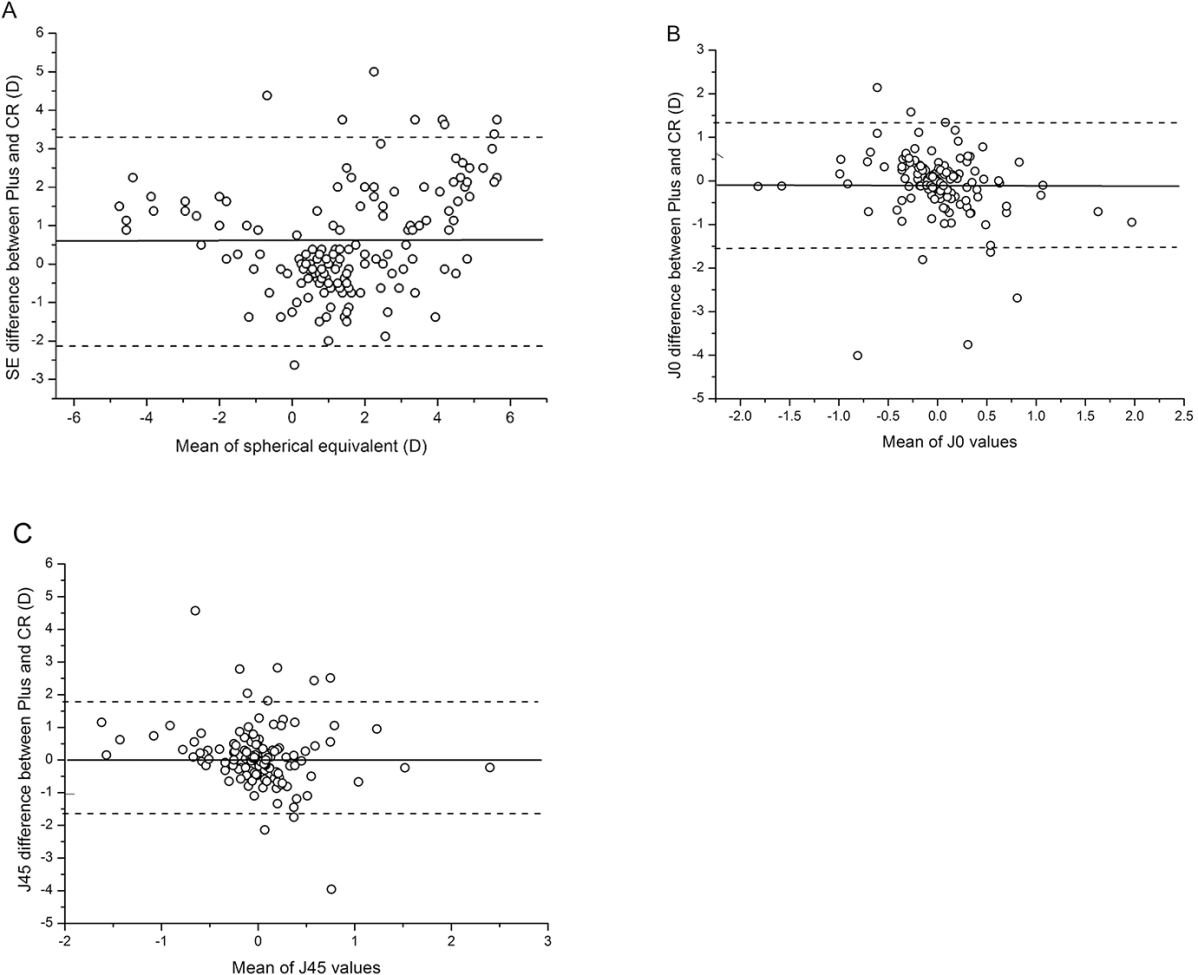
Bland-Altman plots showing agreement between the Plusoptix A09 (Plus) and cycloplegic retinoscopy (CR) for SE, J_0_ and J_45_ values.

The SE obtained from the Plusoptix A09 photoscreener (P_SE_) was 0.57 D lower than that of cycloplegic retinoscopy (CR_SE_). Thus, the Plusoptix A09 photoscreener underestimated the hyperopia and overestimated myopia. The difference between the P_SE_ and CR_SE_ was 0.57 ± 1.40 D (mean ± SD). The difference (CR_SE_—P_SE_) was plotted against the average values [(CR_SE_ + P_SE_)/2] in Fig 2A, which shows that the 95% limits of agreement (LOA) ranged from -2.17 D to +3.31 D. The values (CR_SE_—P_SE_) of 86 (57.3%) cases were within ± 1.0 D. Meanwhile, we found that the difference of J_0_ between the Plusoptix A09 (P_J0_) and cycloplegic retinoscopy (CR_J0_) was equivalent to -0.09 ± 0.73 D (mean ± SD), and for J_45_ was 0.08 ± 0.89 D (mean ± SD), respectively. The 95% LOA of J_0_ was -1.52 D to +1.34 D, while that of J_45_ was -1.66 D to +1.82 D. The differences of cylinder power (CR_J—_P_J_) against their average values [(CR_J_ + P_J_)/2] were plotted. J_0_ of 91.3% of the subjects and J_45_ of 84.0% of the subjects were within ±1.0 D, which indicated a good agreement between the Plusoptix A09 photoscreener and cycloplegic retinoscopy regards cylinder values (Fig 2B and 2C).

### Quantitative relation between the Plusoptix A09 and cycloplegic retinoscopy

**Fig 3:**
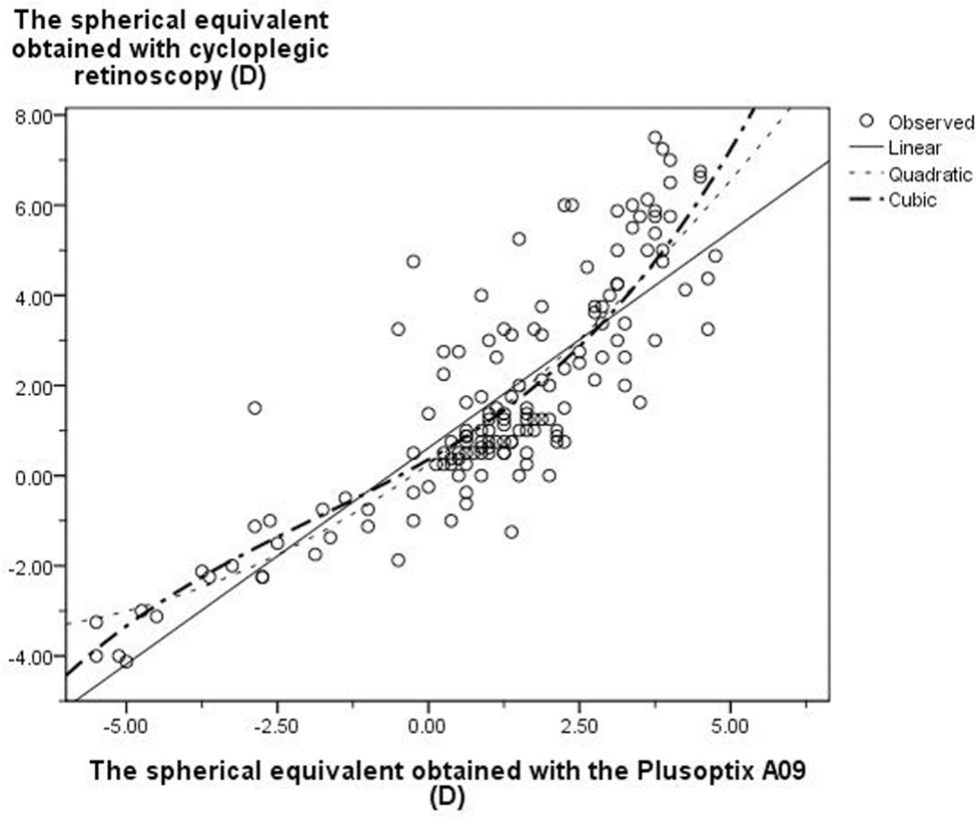
The relationship between the results of the Plusoptix A09 and cycloplegic retinoscopy (three types of custom regression models including linear, quadratic and cubic regression).

Curve estimation regression was used to evaluate the quantitative relationship between the results of the Plusoptix A09 and cycloplegic retinoscopy. Eleven curve models provided by SPSS software were analyzed. Significant differences were observed in three custom regression models (linear, quadratic, and cubic regression) (*P* = 0.00). The coefficient of determination (R^2^) was used to determine the close relationship of variables in the curve estimation regression. Thus, the cubic model, which had a highest R^2^ (0.73), was used as the optimized equation to show the relationship of SEs obtained from the Plusoptix A09 and those of cycloplegic retinoscopy (Fig 3). The cubic regression model is Y = 0.358 + 0.776X + 0.064X^2^ + 0.011X^3^. Clinically, the ‘X’ and ‘Y’ represented P_SE_ and CR_SE_, respectively, i.e. CR_SE_ = 0.358 + 0.776P_SE_ + 0.064 P_SE_^2^ + 0.011 P_SE_^3^.

### Sensitivity and specificity of detecting refractive amblyopia risk factors

Twenty-eight cases, in which the data was reported as “Hyperopia” or “Myopia”, were included in this analysis because they met the referral criteria of the AAPOS.

**Fig 4:**
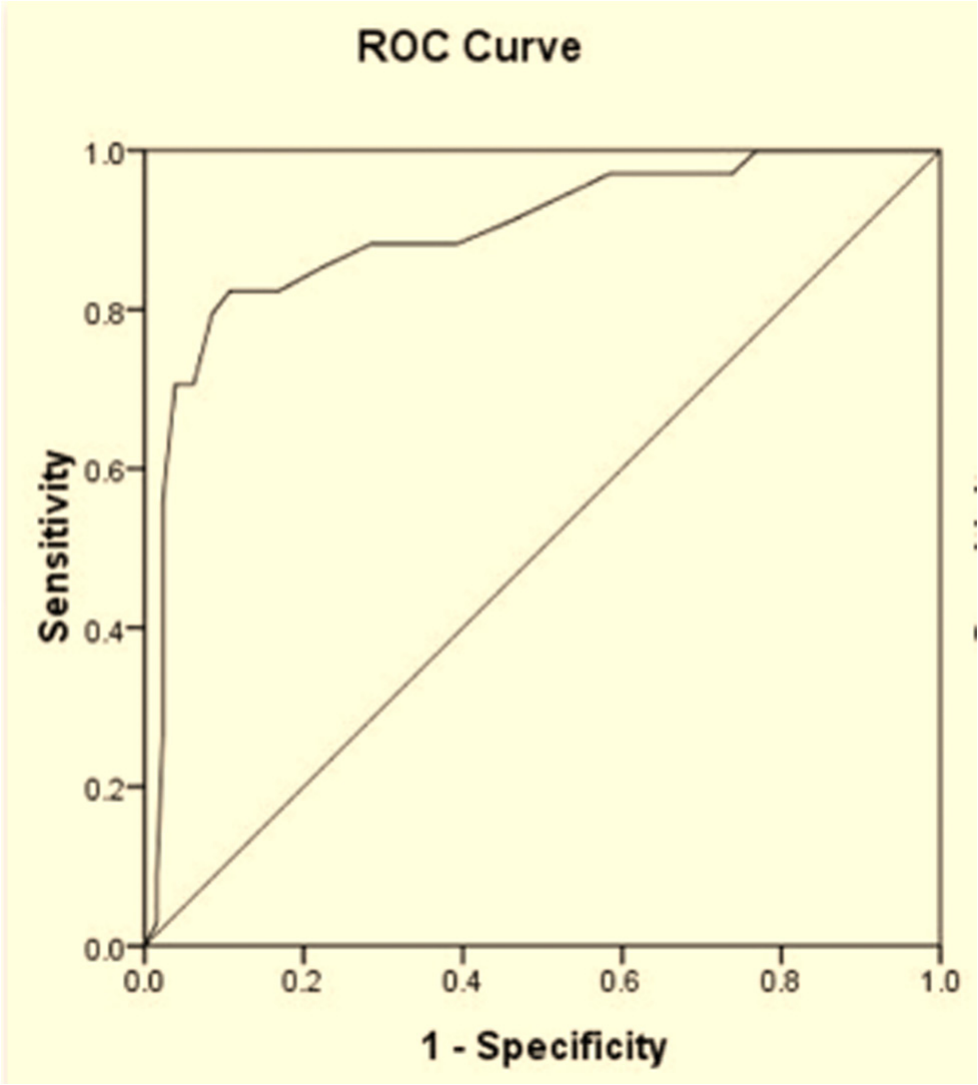
ROC curve for hyperopia obtained by the Plusoptix A09 in comparison with cycloplegic retinoscopy.

**Fig 5:**
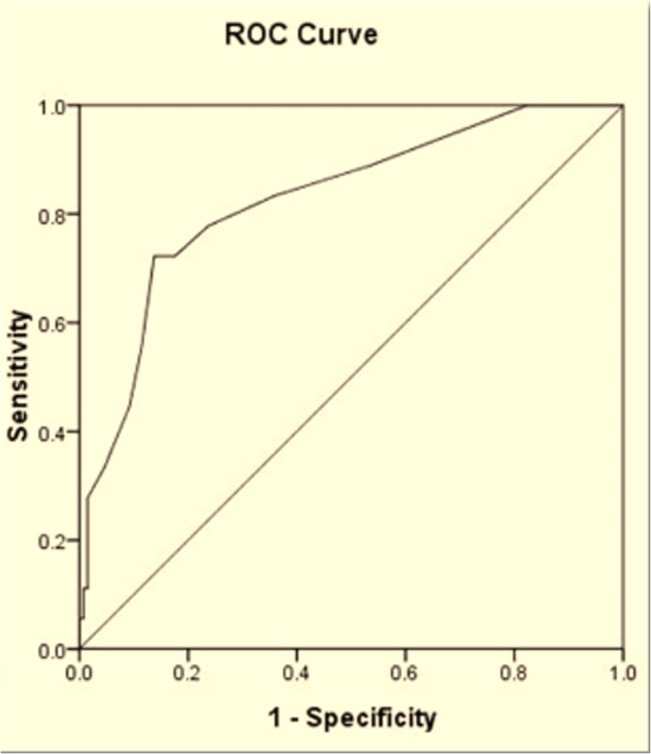
ROC curve for anisometropia obtained by the Plusoptix A09 in comparison with cycloplegic retinoscopy.

In the present study 55.1% of the patients had refractive amblyopia risk factors. The Plusoptix A09 had a sensitivity of 80.6% and specificity of 76.3% in detecting the refractive amblyopia risk factors. The sensitivity and specificity of the Plusoptix A09 in detecting refractive errors according to the AAPOS criteria are shown in Table 2. The sensitivities of the Plusoptix A09 in detecting hyperopia>+3.5 D and anisometropia were low. The ROC curve was used to compare the Plusoptix A09 and cycloplegic retinoscopy in hyperopia and anisometropia. The areas under the curves were 0.90 and 0.83, respectively. The optimal cutoff for hyperopia was +1.88 D and 1.38 D for anisometropia (Figs 4 and 5). According to the ROC curves, the referral criteria for hyperopia and anisometropia were adjusted to +1.88 D and 1.25 D, respectively. Therefore, the overall sensitivity and specificity of the Plusoptix A09 in detecting refractive amblyopia risk factors improved to 94.9% and 67.5%, respectively (Table 3).

**Table 2:** Sensitivity and specificity for detecting refractive amblyopia risk factors with the Plusoptix A09 according to AAPOS.

AAPOS criteria	Hyperopia>3.5 D in any meridian	Myopia>-3.0 D in any meridian	Astigmatism (>1.5 D within 10°of 90°or 180°,>1.0 D in oblique axis)	Anisometropia >1.5 D (spherical or cylindrical)
Sensitivity	44.4%	85.7%	85.0%	55.5%
Specificity	97.7%	94.7%	85.5%	87.9%

**Table 3:** Sensitivity and specificity for detecting refractive amblyopia risk factors with the Plusoptix A09 according to revised criteria on the basis of receiver operating characteristic (ROC) curve.

Revised criteria	Hyperopia>1.88 D in any meridian	Myopia>-3.0 D in any meridian	Astigmatism (>1.5 D within 10°of 90°or180°,>1.0 D in oblique axis)	Anisometropia >1.25 D (spherical or cylindrical)
Sensitivity	86.7%	85.7%	85.0%	72.2%
Specificity	89.5%	94.7%	85.5%	84.8%

### Detection of strabismus

Strabismus was observed in 59 children. The sensitivity and specificity of the Plusoptix A09 in detecting strabismus were 40.7% and 98.3%, respectively. Twenty-one (55.3%) esotropia and four (22.2%) exotropia were screened out using the Plusoptix A09. The other 34 patients were false negatives, including esotropia (n = 17, 16 of them were less than 20 PD), exotropia (n = 5, 4 of them were less than 20 PD) and all of the intermittent exotropia (n = 9) and vertical strabismus (n = 3).

### Sensitivity and specificity of detecting amblyopia and/or strabismus

**Fig 6:**
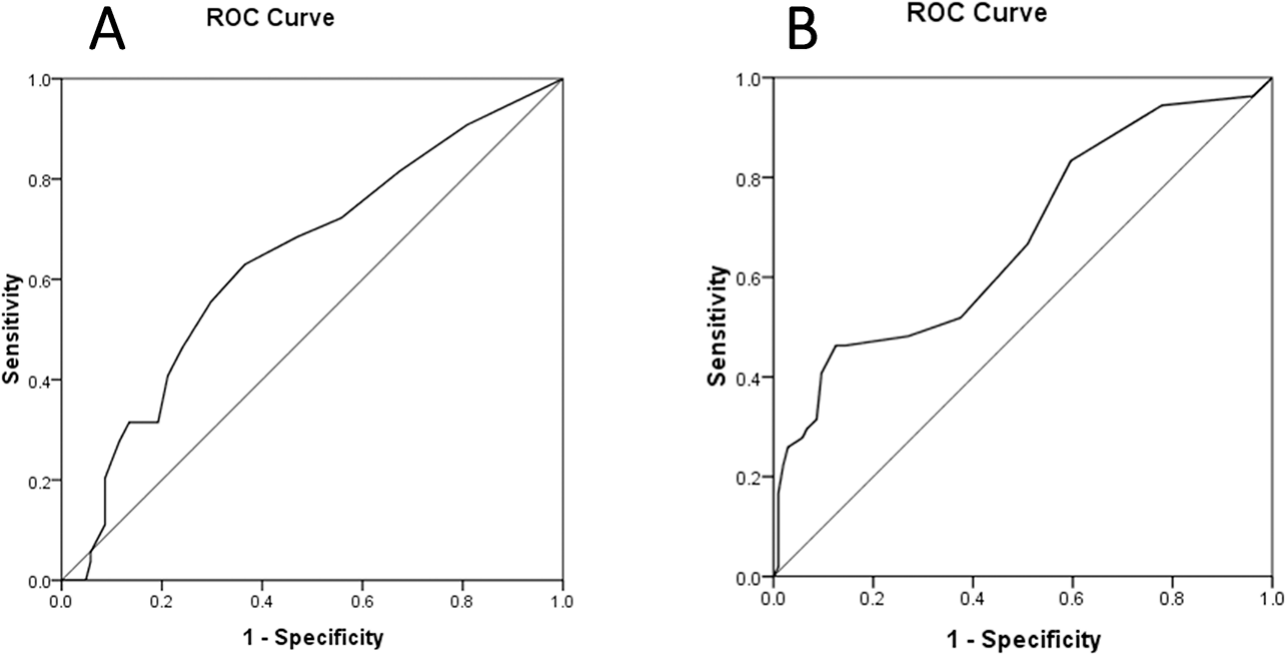
ROC curves of spherical and cylinder values obtained from the Plusoptix A09 in detecting amblyopia.

The sensitivity and specificity of Plusoptix A09 for detection of amblyopia and/or strabismus were 84.7% and 63.2%, respectively. Thirty-nine children had risk factors of developing amblyopia based on the measurements of Plusoptix A09. However, they did not develop amblyopia and/or strabismus and the results from the Plusoptix A09 were considered as false-positives. In Fig 6, ROC curves for detection of amblyopia were generated. The spherical and cylinder values were obtained from the Plusoptix A09, and the areas under the curves were 0.64 and 0.68, respectively.

## Discussion

The present study provides additional information regarding the performance of the Plusoptix A09 photoscreener in children. Compared to the conventional cycloplegic retinoscopy, the Plusoptix A09, an updated version of the Plus photoscreeners, has indispensable merits for the large-scale vision screening. As examples, it is a portable instrument without connection to a laptop computer, has faster data acquisition, is patient-friendly using a smile face with flashing lights as the fixation target, etc… [27]. While cycloplegic retinoscopy is time consuming, uses cycloplegic eye drops, and requires more optometrists, it is not an optimal approach for amblyopia risk factor screening.

In the present study, the Plusoptix A09 is revealed to have a general trend towards myopic values. Specifically, it underestimates hyperopia and overestimates myopia using SE as values, which is in accordance with the reports of the Plusoptix photoscreeners [16–17, 19–21]. Nevertheless, the diopters shifting to myopia were variable in different reports. Moghaddam *et al*., [20] reported that the mean difference of SE measured between the Plusoptix and via retinoscopy was 0.16 D on children aged 6 months to 3 years. Erdurmus’s study [28] included a cohort of healthy children (age: 7.1 ± 2.4 years (mean±SD); range, 9 months to 14 years) and showed that the difference of SE between the Plusoptix and cycloplegic retinoscopy was 0.70 D. Dahlmann-Noor *et al*., [29] recruited 126 children with a mean age of 5.5 years attending hospital-based pediatric eye service to their study, and confirmed a myopic shift of 1.90 D. We have analyzed the relationship of age and myopic shift using regression analysis and observed no significant relationship (P = 0.53). However, it is still early to claim that there is no relationship between age and myopic shift considering the limitation of the present study. For example, the sample size and subjects from an eye clinic may not represent the healthy population. Further study with a large-scale healthy population is warranted.

In addition, there was no significant difference of the mean cylinder value between the Plusoptix A09 and cycloplegic retinoscopy, which is consistent with the previous studies [17, 21]. Bland-Altman plots also showed a poor agreement of SEs and a better agreement of astigmatic measurements between the Plusoptix A09 and cycloplegic retinoscopy. Our results are in agreement with the results reported by Paff *et al*., [19] and Dahlmann-Noor *et al*., [29]. We attribute the poor agreement of SE to the accommodative spasm, which may be induced by staring at the target of the Plusoptix A09 at approximately one-meter distance, since this accommodation was totally diminished in cycloplegic retinoscopy.

Rajavi *et al*., [16] and Erdurmus *et al*., [28] studied the relationship between the Plusoptix photoscreener and cycoplegic retinoscopy by means of Pearson correlation. However, in the present study, we found that both linear regression and quadratic and cubic regressions had significant coefficients between the Plusoptix photoscreener and cycoplegic retinoscopy. Furthermore, compared to linear and quadratic regressions, the cubic model had the highest coefficient of determination. Applying the cubic model presented a more precise relationship between the measurements of the Plusoptix A09 and cycloplegic retinoscopy. To a certain extent, the cubic formula rectified the original results obtained from the Plusoptix A09 and more closely approximated the data obtained via cycloplegic retinoscopy. The accuracy of the Plusoptix photoscreener was controversial because the refractive result of the Plusoptix was not consistent with that of cycloplegic retinoscopy [17, 29, 30]. In the present study, a conversion for the results of either measurement between the two refraction methods was generated. Using the equation established the measurements of the cycloplegic retinoscopy or the Plusoptix A09 photoscreener could be predicted by each other more accurately.

Results in the present study demonstrate that the sensitivity and specificity of the Plusoptix A09 in detecting refractive amblyopia risk factors varied with the selected referral criteria, similar to previous reports [31–33]. We found that it is essential to optimize the referral criteria for the Plusoptix A09 before using it as a screening device for refractive amblyopia risk factors. Singman *et al*., [33] evaluated the sensitivity and specificity of the Plusoptix photoscreener on the same cohort of children by using seven different referral criteria [2, 14, 15, 31, 34–36]. They suggested that vision screening programs should adjust referral criteria according to the local conditions (Table 4). Considering the severe harmfulness of amblyopia and the cost of screening examinations, we prefer an approach with the ability to detect all amblyopia patients in the screening and referral criteria with a higher sensitivity without sacrificing too much specificity. In the present study, we documented a revised referral criteria for the Plusoptix A09, i.e., hyperopia > +1.88 D, myopia > -3.0 D, astigmatism (> 1.5 D within 10°of 90°or 180°, > 1.0 D in oblique axis), or anisometropia > 1.25 D (spherical or cylindrical).

**Table 4:** Sensitivity and specificity of the Plusoptix photoscreener in detecting refractive amblyopia risk factors with various referral criteria.

	Referral Criteria	Sensitivity	Specificity
Plus manufacturer [34]	6–12 months Hyperopia≥3.0D, Myopia≥2.0D, Astigmatism≥1.0D, Anisometropia≥1.0D	98%	41%
	12–36 months Hyperopia≥1.0D, Myopia≥2.0D, Astigmatism≥0.75D, Anisometropia≥1.0D		
	36–7 2months Hyperopia≥1.0D, Myopia≥1.0D, Astigmatism≥0.75D, Anisometropia≥1.0D		
	72–240 months Hyperopia≥0.75D, Myopia≥0.75D, Astigmatism>0.75D, Anisometropia≥0.75D		
AAPOS [2]	Hyperopia>3.5D, Myopia>3.0D, Astigmatism>1.5D or >1.0D oblique axis, Anisometropia>1.5D	74%	86%
Arthur [14]	Hyperopia>3.5D, Myopia>3.0D, Astigmatism>1.25D, Anisometropia>1.5D	81%	92%
Arthur2 [31]	Hyperopia≥3.5D, Myopia≥3.0D, Astigmatism≥2.5D, Anisometropia≥1.5D	67%	96%
Arnold [35]	0–8 months Hyperopia≥3.0D, Myopia≥3.0D, Astigmatism≥2.0D, Anisometropia≥1.5D	81%	96%
	9–72 months Hyperopia≥2.5D, Myopia≥2.25D, Astigmatism≥2.0D, Anisometropia≥1.0D		
	73–120 months Hyperopia≥2.0D, Myopia≥1.5D, Astigmatism≥1.5D, Anisometropia≥1.25D		
PediaVision [36]	6–12 months Hyperopia≥3.5D, Myopia≥2.0D, Astigmatism≥2.25D, Anisometropia≥1.5D	80%	94%
	12–36 months Hyperopia≥3.0D, Myopia≥2.0D, Astigmatism≥2.0D, Anisometropia≥1.0D		
	36–72 months Hyperopia≥2.5D, Myopia≥1.5D, Astigmatism≥1.5D, Anisometropia≥1.0D		
	72–240 months Hyperopia≥2.5D, Myopia≥0.75D, Astigmatism>1.5D, Anisometropia≥1.0D		
	240–1200 months Hyperopia≥1.5D, Myopia≥0.75D, Astigmatism>1.5D, Anisometropia≥1.0D		
Matta/Silbert [15]	6–12 months Hyperopia≥3.0D, Myopia≥2.0D, Astigmatism≥1.0D, Anisometropia≥1.25D	98%	80%
	12–36 months Hyperopia≥1.25D, Myopia≥2.0D, Astigmatism≥1.0D, Anisometropia≥1.25D		
	36–72 months Hyperopia≥1.25D, Myopia≥1.0D, Astigmatism≥1.0D, Anisometropia≥1.25D		
	72–240 months Hyperopia≥1.0D, Myopia≥1.0D, Astigmatism>1.25D, Anisometropia≥1.25D		

In the present study, we found that the sensitivity of the Plusoptix A09 in detecting strabismus was low, especially intermittent exotropia, vertical strabismus, and strabismus with deviation less than 20 PD. The result was similar to published reports [22, 23]. As an ideal screening instrument for amblyopic strabismus, we are looking for a system that could detect strabismus with not only large but also small deviations, since small deviations cannot be easily observed by parents or children themselves. Therefore, further improvement on sensitivity of detecting all kinds of strabismus using the Plusoptix A09 is needed.

A variety of vision screening equipments are mainly used to detect amblyopia and strabismus at an early age when they can be treated. In the present study, the data showed that the Plusoptix A09 had a sensitivity of 84.7% and specificity of 63.2% for detection of amblyopia and/or strabismus. Jost *et al*. [37] performed a study to compare the diagnostic accuracy of a binocular retinal birefringence scanner (PVS) and a SureSight Autorefractor on detection of strabismus and amblyopia. They reported the sensitivity and specificity of the SureSight Autorefractor to be 74% and 62%, respectively, which is similar to the results of our study. Moreover, their findings supported that the PVS, with the sensitivity of 97% and specificity of 87%, outperformed the SureSight Autorefractor in identifying amblyopia and/or strabismus. Schmidt *et al*. [38] performed a comparison among 11 preschool vision screening tests and provided sensitivities of each test with the specificity set as 90% for detection of amblyopia, strabismus and significant refractive errors. When comparing our results to the study by Schmidt *et al*. [38], it is important to point out that the Plusoptix photoscreener A09 has higher sensitivity in detecting amblyopia and refractive amblyopia risk factors in children (Table 5).

**Table 5:** Sensitivity and specificity of different vision screening techniques in detecting amblyopia, strabismus and refractive amblyopia risk factors.

Vision Screeners	Amblyopia		Strabismus		Refractive risk factors	
	Sensitivity	Specificity	Sensitivity	Specificity	Sensitivity	Specificity
Lea Symbols test [38]	0.76	0.90	0.56	0.90	0.70	0.90
HOTV VA Test [38]	0.73	0.89	0.65	0.89	0.59	0.89
Random Dot E stereoacuity [38]	0.63	0.90	0.60	0.90	0.47	0.90
Noncycloplegic retinoscopy [38]	0.85	0.90	0.56	0.90	0.81	0.90
MTI photoscreener [38]	0.63	0.94	0.65	0.94	0.42	0.94
The Plusoptix A09	0.85	0.63	0.41	0.98	0.95	0.68

There is a limitation of the present study. Since the subjects involved were patients attending our eye clinic, the results may be affected by a higher prevalence of eye diseases than those in a healthy population. A population based large-scale photorefraction in a normal child population is underway to further substantiate the results obtained herein.

In conclusion, compared with cycloplegic retinoscopy, the Plusoptix A09 underestimates hyperopia and overestimates myopia based on SE. The regression equation and optimized criteria for amblyopia risk factors established in the present study can improve the accuracy of the Plusoptix A09 in amblyopia risk factor screening in children. The Plusoptix A09 has various advantages including time saving, easily manipulated, and good compliance of targeted children. Thus, compared with other photoscreener instruments and retinoscopy examination, the Plusoptix A09 is an acceptable technique for large-scale refraction examination in children using appropriate referral criteria. However, the Plusoptix A09 photoscreener is not suitable for large-scale strabismus screening solely.
